# Characterization of Novel Variants in *P2YRY12, GP6* and *TBXAS1* in Patients with Lifelong History of Bleeding

**DOI:** 10.3390/biom15121639

**Published:** 2025-11-21

**Authors:** Ana Zamora-Cánovas, Ana Marín-Quílez, Lorena Díaz-Ajenjo, Ana Sánchez-Fuentes, Pedro Luis Gómez-González, Marilena Crescente, Nuria Fernández-Mosteirín, José Padilla, José Ramón González-Porras, Rocío Benito, María Luisa Lozano, José María Bastida, José Rivera Pozo

**Affiliations:** 1 Servicio de Hematología, Hospital Universitario Morales Meseguer, Centro Regional de Hemodonación, Universidad de Murcia, IMIB-Pascual Parrilla, CIBERER-ISCIII, 30003 Murcia, Spainana.marin.94@gmail.com (A.M.-Q.); ana.sanchez1610@gmail.com (A.S.-F.); pedrol.gomez@imib.es (P.L.G.-G.);; 2 Instituto de Investigación Biomédica de Salamanca (IBSAL), Centro de Investigación del Cáncer, IBMCC-CSIC, Universidad de Salamanca (USAL), 37007 Salamanca, Spain; lorenadiaj21646@usal.es (L.D.-A.); beniroc@usal.es (R.B.); jmbastida@saludcastillayleon.es (J.M.B.); 3 Centre for Immunobiology, Faculty of Medicine and Dentistry, Blizard Institute, Queen Mary University of London, London E1 2AD, UK; 4 Servicio de Hematología, Hospital Universitario Miguel Servet, 50009 Zaragoza, Spain; nfernandezm@salud.aragon.es; 5 Departamento de Hematología, Complejo Asistencial Universitario de Salamanca (CAUSA), 37007 Salamanca, Spain

**Keywords:** inherited platelet function disorders, IPFD, P2Y12 deficiency, GPVI deficiency, TBXAS1 deficiency, bleeding

## Abstract

Inherited platelet function disorders (IPFDs) are rare diseases caused by defects in platelet surface receptors, enzymes, granules, or signaling proteins. In humans, GPVI and P2Y12 deficiency cause autosomal recessive bleeding disorders, while TBXAS1 deficiency is related to Ghosal hematodiaphyseal dysplasa, a rare autosomal recessive disorder characterized by increased long bone density and platelet dysfunction without bleeding. To date, at least 20 patients have been identified with molecular defects in *P2RY12*, 12 cases with molecular defects in *GP6*, and 34 cases with molecular defects in *TBXAS1*. Here, we report a novel nonsense and missense variants in *P2RY12*, a novel nonsense variant in *GP6*, and a novel missense variant in *TBXAS1*. These variants selectively affect the platelet reactivity to ADP and collagen/CRP, predisposing to bleeding. *P2RY12* c.835 G>A [p.Val279Met] variant did not affect receptor expression whereas *P2RY12* c.44delG [p.Ser15Ilefs*33] lead to decreased levels of the receptor in one of the patients. This was confirmed both by RT-qPCR and immunoblotting analysis. Decreased expression of both GPVI and FcRγ-chain was detected in patients carrying GPVI nonsense variant in heterozygosis. The deleterious effect of these variants was also confirmed in a transfected cell line model. *TBXAS1* variant triggered decreased TxA_2_ production using a cell line model. These variants expand the genetic landscape of P2RY12, GPVI and TBXAS1 inherited deficiency.

## 1. Introduction

Inherited platelet disorders (IPDs) comprise a heterogeneous group of rare diseases, generally caused by molecular alterations in genes that are relevant in platelet formation and/or function [1]. Two main groups of IPDs can be distinguished as follows: inherited thrombocytopenias (IT), characterized by low platelet counts, and inherited platelet function disorders (IPFD), in which patients display normal platelet counts but altered platelet function [2,3]. The risk of bleeding is a common characteristic of these types of disorders [4].

The diagnosis of IPDs is limited due to their high clinical and laboratory heterogeneity, as well as the poor reproducibility and specificity of platelet function tests [1]. Therefore, expert guidelines recommend different approaches for its diagnosis [5,6]. Firstly, a comprehensive clinical assessment is usually performed by exploring different signs of bleeding and discussing family history [6]. This is usually followed by a laboratory study with different standardized techniques, including blood count and smear, semi-automated screening assays, such as Platelet Function Analizer-100 assay (PFA-100), and other analyses of platelet function [7]. Later, genetic diagnosis helps to outline the precise cause of IPDs, especially when other laboratory tests are not informative [6,8].

Sanger sequencing has long been the standard assay used to discern the candidate variants [9]. However, we were only able to achieve genetic diagnosis in 40% of cases suspected to have an inherited platelet disorder using this candidate gene approach [10]. The recent incorporation of high-throughput sequencing (HTS) has enhanced the genetic diagnosis of IPDs, with many specialized laboratories now implementing this approach [11,12]. Using this novel approach, we achieved a genetic diagnosis rate of 70% [12]. In the largest series of cases described so far, the diagnostic rate was 47.8% for inherited thrombocytopenia, 26.1% for platelet dysfunction, and 63.6% for coagulation disorders [11]. Moreover, the advent of third generation sequencing techniques, such as Nanopore sequencing, allows long-read sequencing of DNA and characterization of structural variants (SVs) [13].

The specificity of a clinical phenotype provides critical information for the interpretation of genetic data and determining clinical significance [8]. This enables researchers to establish relationships between platelet phenotype and genotype, since an early phenotypic and genetic diagnosis is crucial for effective patient management [6,14].

IPFD are heterogeneous in severity, mechanisms, and frequency, and few are characterized at the molecular level. To date, roughly 20 different disorders have been described [3]. These are caused mainly by defects of the membrane receptors (Glanzmann thrombasthenia, P2Y12 deficiency, glycoprotein VI (GPVI) deficiency, among others), granules (Hermansky–Pudlak syndrome, among others), elements involved in signal transduction, or other defects of the biochemical platelet machinery (such as thromboxane A synthase 1 (TBXAS1) deficiency) [15].

Here we present a comprehensive review of the IPFD affecting the adenosine diphosphate (ADP) receptor P2Y12, the collagen receptor GPVI, and TBXAS1. This will be followed by a brief description of three patients with two novel heterozygous variants in *P2RY12*, two patients with a novel heterozygous nonsense variant in *GP6* and finally, a novel heterozygous variant in *TBXAS1*. Clinical evaluation and platelet phenotyping assays were performed to assess the effect of all variants. Finally, the deleterious effect was also evaluated in a cell line model transfected with wild-type proteins and all variants.

### 1.1. P2Y12-Related Disorder

The P2Y12 receptor gene (*P2RY12*), encodes a major G-protein-coupled receptor (GPCR) in platelets that maps to chromosome 3q25.1. *P2RY12* contains three exons. The receptor consists of 342 amino acids with a molecular weight of 39 kDa.

**Figure 1:**
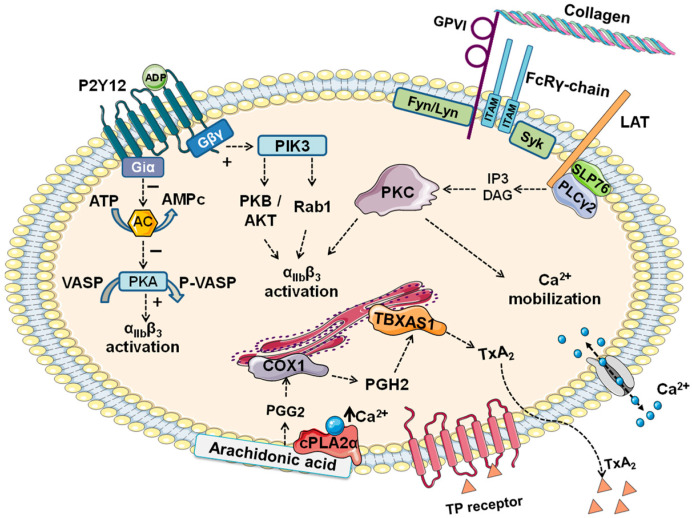
Schematic representation of P2Y12 (top left), GPVI (top right), and TBXAS1 (bottom) pathways. **Top left**: ADP binds to the receptor, triggering G protein subunit alpha i2 (Gαi2) protein activation. This leads to inhibition of adenylate cyclase, decreasing cyclic adenosine monophosphate (cAMP) production. Thus, protein kinase A (PKA) activity decreases, leading to inactivation of the downstream effector vasodilator-stimulated phosphoprotein (VASP). On the other hand, activation of βγ subunits of Gi promotes activation of phosphoinositide 3-kinase (PI3K). Finally, both Ras-related protein (Rap1b) and protein kinase B (PKB/Akt) contribute to integrin αIIbβ3 (fibrinogen receptor) activation and stabilization of platelet aggregation. **Top right**: Binding to collagen triggers clustering of GPVI with FcRγ-chain, promoting tyrosine-protein kinases Lyn and Fyn phosphorylation of the immunoreceptor tyrosine-based activation motifs (ITAM). These events lead to the formation of a signalosome due to the recruitment of LAT and SLP-76 kinases. Furthermore, activation of phospholipase C (PLCγ2) promotes diacylglycerol (DAG) and inositol triphosphate (IP3). These messengers mediate Protein kinase C (PKC) activation and Ca^2+^ mobilization. **Bottom**: Intracellular Ca^2+^ levels increase, activating phospholipase A2 (cPLA2α), releasing arachidonic acid (AA) from the membrane. Then, Prostaglandin G2 (PGG_2_) is processed into Prostaglandin H2 (PGH_2_) by the action of cicloxigenase-1 (COX1). Afterwards, thromboxane A synthase 1 (TBXAS1) catalyzes the conversion to thromboxane A_2_ (TxA_2_). The metabolite binds to the TxA_2_ receptor (TP) on the membrane, triggering Ca^2+^ mobilization promoting platelet aggregation. Image was drawn adapting images provided by Servier Medical Art (https://smart.servier.com/, accessed on 21 October 2025), licensed under CC BY 4.0 (https://creativecommons.org/licenses/by/4.0/, accessed on 21 October 2025).

Binding to ADP triggers G protein subunit alpha i2 (Gαi2) protein (associated with P2Y12) activation, leading to the inhibition of adenylate cyclase (AC), and therefore cyclic adenosine monophosphate (cAMP) production [16] (Figure 1). This inhibition impairs protein kinase A (PKA) activity and subsequent activation of downstream effectors such as vasodilator-stimulated phosphoprotein (VASP) [17] (Figure 1). This pathway is not sufficient to start platelet aggregation, but it can promote platelet activation. The receptor also leads to the activation of the phosphoinositide 3-kinase (PI3K) pathway, through the mediation of G protein subunit βγ (Giβγ). This mechanism promotes integrin αIIbβ3 (fibrinogen receptor) and stabilization of platelet aggregation [18] (Figure 1). Therefore, co-activation of both P2Y1 and P2Y12 is required for normal ADP-induced platelet aggregation [19].

Different roles for P2Y12 have been described. P2Y12 activation is thought to induce an inflammatory state in vascular smooth muscle cells (VSMC), promoting atherosclerotic plaque instability [20]. But also, P2Y12 has a role in microglial cell activation, as well as communication with neurons [21]. Finally, other studies have proposed P2Y12 implication in the purinergic signaling in leukocytes [22].

Moreover, P2Y12 has been used as a target for various treatments. Several clinical antiplatelet drugs (copidrogel and ticagrelor) have been designed as antagonists of the P2Y12 receptor [23]. The use of these inhibitors has shown in patients with acute coronary syndrome (ACS) a decrease in the risk of thrombotic events.

P2Y12 deficiency (OMIM #609821) caused an autosomal recessive bleeding disorder. This deficiency is characterized by decreased P2Y12 expression, mild to moderate mucocutaneous bleeding, and excessive bleeding in response to ADP and trauma or after surgery [24].

**Figure 2:**
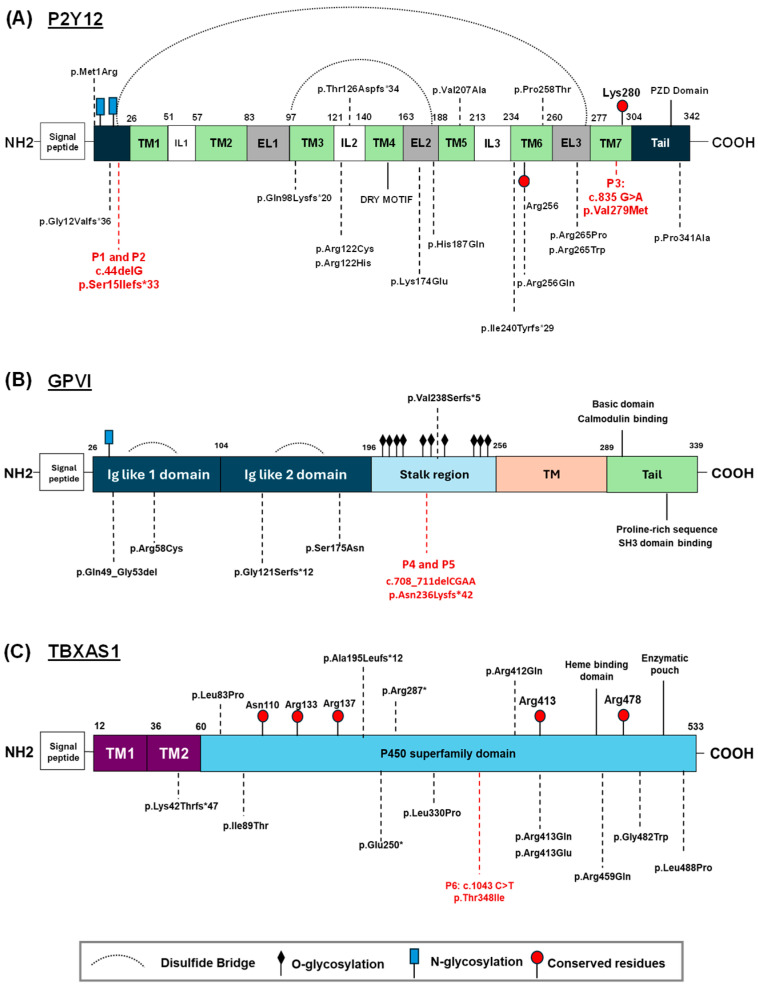
Schematic representation of P2Y12, GPVI, and TBXAS1 structures and domains. (**A**) Localization of the *P2RY12* variants presented in all probands (dotted red lines), as well as all previously reported variants (black dotted lines). Its structure comprises seven transmembrane α-helices (TM), connected by three intracellular (IL), and three extracellular loops (EL). Two potential N-linked glycosylation sites and two disulfide bridges have been identified. Two highly conserved residues (Arg256 and Lys280) are predicted to bind to ADP. (**B**) Localization of novel *GP6* frameshift variant (red dotted line) as well as all previously reported variants (black dotted lines). The extracellular part contains two Ig-like domains (D1 and D2). These domains are followed by a highly O-glycosylated mucin-like region. GPVI also contains different sites of action of protease ADAM10, which allow cleavage of its extracellular portion in response to different stimuli. (**C**) Localization of novel missense variant in TBXAS1 (red dotted line), as well as previously reported variants (black dotted lines). All relevant residues are highlighted in red circles. N-glycosylation sites are highlighted by blue squares. O-glycosylation sites are highlighted by a dark rhombus. Finally, disulfide bridges are highlighted in black.

To date, at least 20 patients have been identified with molecular defects in *P2RY12,* causing quantitative and qualitative defects in the receptor (Figure 2A, Supplemental Table S1) [25,26,27,28,29,30,31,32,33,34,35,36,37,38] of which six have been described with variants decreasing P2Y12 expression [25,26,27,30,31,34]. It was assumed that heterozygous variant carriers were asymptomatic. However, in one study, the authors described a patient carrying the p.Ile240Tyrfs*29 variant in heterozygosis with reduced P2Y12 expression [34]. These results suggest that the disease associated with defects in P2Y12 can also cause a pathological phenotype in heterozygosis.

### 1.2. GPVI-Related Disorder

The *GP6* gene encodes for the glycoprotein VI (GPVI), and it is located at 19q13.4, within the leukocyte receptor cluster (LCR), and its expression is restricted to platelets and megakaryocytes [39,40]. *GP6* comprises eight exons, while GPVI is a 339 amino acid transmembrane protein (60kDa) with a high presence of carbohydrates (45%).

GPVI belongs to the immunoglobulin-like receptor superfamily and is co-expressed on the platelet surface in a non-covalent bond with the common Fc receptor-γ-chain (FcRγ-chain), which contains the ITAM responsible for intracellular signaling [39,41]. The glycoprotein has been described as the main receptor involved in platelet activation upon interaction with collagen [40]. However, several studies have reported the interaction of GPVI with other vascular wall ligands such as laminin, fibrinogen, fibronectin or vitronectin, or plasma ligands such as the hormone adiponectin and histones [42,43,44,45,46,47]. Apart from GPVI, platelets express two other immune-like receptors, the podoplanin receptor C-type lectin domain family 2 (CLEC-2) and the low-affinity IgG receptor FcγRIIA [48]. All three receptors have in common their role in the transmission of activation signals into the platelet via immunoreceptor tyrosine-based activation motifs (ITAMs) [49].

The GPVI transmembrane region contains critical residues that interact with the FcRγ-chain [50]. Moreover, the cytosolic region of GPVI contains a basic sequence that contributes to this interaction [50,51]. This region also presents a calmodulin-binding motif and a proline-rich domain (PRD) [50,51]. Tyrosine-protein kinases Lyn and Fyn, both Src family kinases, constrictively bind to this domain [52]. Upon interaction of GPVI with its ligands, these kinases phosphorylate the ITAM domains of the GPVI-bound FcRγ-chain homodimer, leading to recruitment and activation of the tyrosine kinase Syk [40] (Figure 1). This triggers new downstream signaling events, with recruitment of new signaling proteins and kinases, such as LAT, SLP-76, and others, forming a potent signalosome, which mediates the activation of phospholipase C (PLCγ2) and the subsequent generation of the second messengers diacylglycerol (DAG) and inositol triphosphate [53]. These messengers mediate Protein kinase C (PKC) activation and Ca^2+^ release from intraplatelet stores, respectively, and ultimately platelet responses to thromboxane A_2_ generation (TxA_2_), secretion, integrin activation αIIbβ3 and aggregation (Figure 1).

Moreover, GPVI transmits outside-in activation signals that contribute to platelet spreading and platelet aggregation under flow conditions [47]. Therefore, GPVI acts as a key element both in the initiation of thrombus formation at sites of vascular injury, as well as in subsequent thrombus growth through the recruitment of new platelets [54].

The role of GPVI in platelet–collagen interactions, and thus in haemostasis, was demonstrated by the development of GPVI knock-out (KO) mice [55,56]. Several studies have demonstrated, using murine models, the importance of the interaction with the FcRγ-chain for GPVI expression on the platelet’s surface [57,58,59]. Platelets from GPVI KO mice show impaired platelet aggregation and significant failure of platelet activation with collagen, collagen-related-peptide (CRP), or the snake venom convulxin, which are selective GPV agonists [55]. In heterozygous mice, hypo-reactivity to collagen is evident at low doses, but recovers at high doses [55,56].

Interestingly, tail bleeding time is minimally prolonged, indicating that the haemostatic role of GPVI can be performed by other platelet elements [56]. This is consistent with the observation of moderate clinical hemorrhage in the few patients with congenital GPVI deficiency identified to date [60]. However, GPVI KO mice without GPVI appear to be protected from thrombosis, in models of experimental thrombosis induced by collagen injection or in models of thrombosis with FeCl_3_ [56,61].

In humans, GPVI deficiency (OMIM #614201) causes an autosomal recessive bleeding disorder. GPVI deficiency, both acquired and congenital, is a rarely described pathology, either because it is infrequent, or because it is associated with an insignificant clinical presentation, and affected patients go unnoticed [62].

Around 10 patients have been described with immune or pseudo-immune conditions associated with quantitative GPVI deficiencies [63]. This was associated with the presence of antibodies that were not always detectable. Almost all cases were female, displayed thrombocytopenia, and responded to treatment with steroids or other drugs used in immune thrombocytopenia (ITP) [62,63].

Regarding inherited GPVI deficiency, a dozen cases have been reported in the literature [54]. Among these, molecular defects in the *GP6* gene were identified in nine cases with five different variants [54] (Figure 2B, Supplemental Table S2). Two cases involve patients with two compound heterozygous mutations, while a small group of unrelated Chilean patients share the same nucleotide insertion that breaks the normal reading phase and results in a truncated GPVI [64]. All evidence suggests a founder effect. The Chilean patients had mild bleeding, while heterozygous family members were asymptomatic [64].

Inherited GPVI deficiency is characterized by impaired response to CRP, collagen, and convulxin. Some of the patients displayed reduced levels of GPVI expression, confirmed both by flow cytometry and Western blot [64]. However, in other cases, the expression levels were normal [65]. Decreased levels of FcRγ-chain were also found in all Chilean homozygous patients [66]. Furthermore, normal levels were reported in heterozygous patients carrying p.Arg58Cys variant [67].

### 1.3. TBXAS1—Related Disorder

Thromboxane synthase (TBXAS1) is a microsomal enzyme found in platelets and several other tissues [68]. It is a cytochrome P450 enzyme that catalyzes the conversion of prostaglandin H2 (PGH_2_) into TxA_2_ [69]. *TBXAS1* locates in chromosome 7q33-34 and contains 17 exons (4 noncoding and 13 coding) [70]. TBXAS1 protein has 533 acids (60 kDa) and contains a heme prosthetic group.

Upon platelet activation, the increase in intracellular Ca^2+^ levels activates phospholipase A2 (cPLA2α), which releases arachidonic acid (AA) from the membrane phospholipids [71]. This substrate is rapidly processed in different pathways, including its conversion TxA_2_ by the sequential actions of cicloxigenase-1 (COX1) and thromboxane synthase [72]. TxA_2_ acts as a potent prothrombotic agent, contributing to platelet aggregation and thrombus formation at sites of vascular injury [73]. Following its release, TxA_2_ bind to the TxA_2_ receptor (TP) on the platelet surface, triggering Ca^2+^ mobilization, protein phosphorylation, secretion and ultimately platelet aggregation [74] (Figure 1).

Ghosal hematodiaphyseal dysplasia (GHDD; OMIM #231095) is a rare autosomal recessive disorder characterized by bone marrow dysfunction and increased long bone density with metadiaphyseal dysplasia [75]. After its initial report [76], roughly 34 more cases have been reported in the literature (Supplemental Table S3). The manifestations of GHDD are present in childhood with bony changes and normocytic anemia often requiring multiple transfusions [77].

GHDD is caused by homozygous or compound heterozygous variants in *TBXAS1* (Figure 2C, Supplemental Table S3). Heterozygous carriers do not develop GHDD symptoms. Although *TBXAS1* alterations were thought to be responsible for a mild bleeding disorder, none of the described patients with GHDD reported bleeding manifestations [75,77,78,79,80,81,82,83,84,85,86,87,88,89,90,91,92,93,94]. Only one of the cases reported in the literature described a patient with gastrointestinal bleeding [95]. However, no molecular diagnosis assays were performed to identify variants in *TBXAS1*. Noteworthy, some patients also presented mild thrombocytopenia (Supplemental Table S3). Platelets from these patients do not aggregate in response to AA, but they respond to the synthetic Tx analog U46619. Furthermore, patients present decreased levels of TxA_2_ in serum [96].

Moreover, it has been described that *TBXAS1* polymorphisms may be relevant for the pharmacogenetics of aspirin and other nonsteroidal anti-inflammatory drugs (NSAIDs), and TxA_2_ has been linked to the cardiovascular toxicity of COX2 specific inhibitors [97].

## 2. Materials and Methods

### 2.1. Patients, Blood Sampling, and DNA Isolation

Six patients with suspected IPFD were recruited in the Spanish multicenter project “Functional and Molecular Characterization of Patients with Inherited Platelet Disorders”. This project obtained approval from the Ethics Committee of the Hospital Reina Sofía (Murcia, Spain) and follows the Helsinki Declaration rules. All participants gave written informed consent. Clinical data were reviewed, and bleeding symptoms were scored using the International Society on Thrombosis and Haemostasis bleeding assessment tool (ISTH-BAT) [98,99].

Venous blood was drawn from each patient into commercial 7.5% K3 ethylenediaminetetraacetic acid (EDTA) tubes (for complete blood count and DNA isolation), and into buffered 3.2% sodium citrate for platelet function studies. Blood counts were performed using a Sysmex^®^ XS1000i hematology counter (Sysmex España, Sant Just Desvern, Barcelona, Spain). Genomic DNA was isolated using a DNeasy blood and tissue kit, following the manufacturer’s protocol (Qiagen, Hilden, Germany). DNA concentration was measured using a fluorometer Qubit 2.0 (Life Technologies, Carlsbad, CA, USA).

### 2.2. Molecular Analysis by HTS Gene Panel and Sanger Sequencing

Patients’ DNA was analyzed by HTS using a panel approach including all exons, 3′ untranslated region and flanking regions of 102 genes relate with IPDs [12]. Samples were sequenced using a MiSeq Illumina platform (Illumina, San Diego, CA, USA), or an Ion Torrent PGM platform (Thermo Fisher Scientific, Waltham, MA, USA). Sequences were aligned to the hg19 reference genome. Variant calling and annotation was performed using an in-house pipeline (VarScan v2.3.9, SAMTools v1.3.1, ANNOVAR (v.20190oct24), Ensembl-VEP v99, and dbNFSP v4.0a bioinformatic tools). Selected variants were confirmed and segregated in the families by Sanger sequencing. General information regarding gene variants (chromosome position, Human Genome Variation Society [HGVS] name, reference single nucleotide polymorphism (SNP) cluster ID number [RS ID], frequency in different populations, pathogenicity and conservation scores, automated classification, ClinVar annotation, etc.) was initially obtained using the Varsome tool (https://varsome.com, accessed on 21 October 2025).

Further analysis of variants was achieved using DIGEVAR ‘’Discovering Genetic Variants’’, a web tool developed in-house for user-friendly analysis of HTS data (https://digevar.imib.es, accessed on 21 October 2025). DIGEVAR was developed in JAVA to access variant calling files (VCF) and allows multiple variant filtering strategies regarding variant gene location, minor allele frequency (MAF), among others. After this selection, DIGEVAR provides the user with a list of candidate variants that match the filtering criteria. As DIGEVAR incorporates information from public databases (ENSEMBL, NCBI, CLINVAR, ExAC, etc.) and from variant analysis software (MutationTaster, Polyphen, Sift, PDB, etc.), it also allows analysis of the allele frequency, nucleotide/protein change, CLINVAR significance, among others.

### 2.3. GP6 and P2RY12 mRNA Quantification by RT-qPCR

Platelet RNA was isolated by using Trizol (Thermo Fisher Scientific, Waltham, MA, USA). *GP6* and *P2RY12* mRNA expressions were measured to investigate the effect of candidate variants on expression levels. cDNA synthesis was performed using the SuperScript IV™ First-Strand cDNA Synthesis System from Invitrogen™, and specific probes for both *GP6* and *P2RY12* were purchased from Thermo Fisher. Procedures were followed as described previously [100].

### 2.4. Platelet Aggregation

Platelet-rich plasma (PRP) and platelet-poor plasma (PPP) were prepared from citrate whole blood by centrifugation (140× *g*, 10 min and 1000× *g,* 10 min, respectively). Light transmission aggregometry (LTA) was performed in PRP (~250 × 10^9^ platelets/L) as previously described [101] using a Stago aggregometer (Stago, Asnieres-sur-Seine, France). Maximum percentages of light transmission of PRP over baseline PPP were recorded for 300 s following stimulation with the following platelet agonists: 25 μM thrombin receptor—activating peptide (TRAP); 2.5, 5, and 10 μM ADP; 1.25 mg/mL ristocetin; 2, 5, and 10 µg/mL collagen; 1.6 mM AA; and 2 and 5 μg CRP.

### 2.5. Platelet Flow Cytometry

Platelet expression of different membrane glycoproteins (GPs), including GPIa (integrin α2, CD49), GPIbα (CD42b), GPIX (CD42a), GPIIb (CD41), and GPIIIa (CD61), and GPVI, were evaluated by flow cytometry in citrated whole blood diluted 1:10 in sterile phosphate-buffered saline (PBS). 

Platelet activation was analyzed by evaluating platelet granule secretion and αIIbβ3 activation as previously described [101]. Briefly, diluted PRP (∼20 × 10^9^ platelets/L) was incubated under static conditions: 30 min at room temperature [RT]) with Tyrode’s buffer, as control for non-stimulated platelets, or with agonists (25 μM TRAP, 2 and 10 μg CRP, 2.5 and 10 μM ADP), in the presence of anti-CD41*APC (as a platelet marker), fibrinogen--Alexa488 (Thermo Fisher) and anti-CD62*PE (α-granule secretion) or anti-CD63*PE (dense granule secretion) (BD Biosciences, Madrid, Spain). Reactions were stopped with 4% paraformaldehyde (PFA) (*v*/*v*) (15 min, RT). Samples were diluted with PBS and run in an Accuri C6 flow cytometer (BD Biosciences). A total of 10,000 platelets were gated on both CD41+ and forward scatter-side scatter (FSC-SSC), and results were expressed as median fluorescence intensity (MFI).

Quantification of receptor molecules (1G5, CD42b, GPVI) per platelet was performed by using a commercial kit (Platelet GP Screen, Biocytex, Stago, Asnières, France).

### 2.6. VASP Phosphorylation Assessment

VASP phosphorylation upon stimulation of the P2Y12 receptor was analyzed by using Biocytex VASP phosphorylation assay (PLT VASP/P2Y12 (Stago). The test was performed according to the manufacturer’s instructions within 48 h of blood collection [102]. The results were expressed as platelet reactivity index (PRI).

### 2.7. Immunofluorescence Studies

The general organization of the platelet cytoskeleton was evaluated by immunofluorescence assays. Washed platelet samples were placed onto coverslips on a 24-well plate under spreading conditions, as previously described [100]. Briefly, platelets were allowed to adhere and to spread in two different matrices of fibrinogen and collagen for 10 and 30 min, and, after fixation with 4% PFA, platelets were visualized with fluorochrome-labeled phalloidin antibodies (Phalloidin–Atto 647N Merck, 1:100) on a Leica SP8 (Leica Microsystems, Madrid, Spain). Spreading of platelets was calculated by analyzing platelets present in 10 different images.

### 2.8. Cell Line Models

The effect of the *P2RY12*, *TBXAS1*, and *GP6* variants was evaluated in HEK 293T cells. Additionally, a HEK 293 [100] in-house cell line with a stable expression of COX-1 wild-type (HEK 293 COX1), previously described [101], was also used for the study of the *TBXAS1* variant. The wild-type HEK 293 cell line does not express P2Y12 and GPVI receptors, albeit they express low levels of TBXAS1.

Transient expression experiments were performed using pcDNA3.1+/C-(K)-DYK with complementary DNA (cDNA) wild-type of *P2RY12*, *TBXAS1* and *GP6*, or mutants, all of them available commercially (OHu14933D, GenScript, Rijswijk, The Netherlands).

HEK 293 cell line (ATCC, LGC Standards S.L.U, Barcelona, Spain) was grown in Dulbecco’s modified Eagle’s medium (DMEM) supplemented with 10% of fetal bovine serum (FBS). Transient expression assays were performed by plating 7 × 10^4^ cells/well and adding all 2 µg/well of all plasmids using Lipofectamine 3000 kit (Thermo Fisher Scientific). Transfection efficiency was assessed by flow cytometry using BD IntraSure (BD Biosciences) and the antibody α-DYKDDDDK*PE (BioLegend, San Diego, CA, USA, Cat#637309). Cell lysates were prepared 48 h after successful transfection for immunoblotting assays. Moreover, 30 × 10^4^ cells/vector transfected were stimulated either with PBS or AA 2 μM (37 °C, 30 min, 500 rpm in a shaker). Samples were centrifuged 1000× *g* for 3 min and supernatants were collected to be stored at −80 °C for TxA_2_ determination (TxB_2_ ELISA kit, Cayman Chemical, Ann Arbor, MI, USA), as described [101]. Statistical analysis was performed using GraphPad Prism 10.0 (San Diego, CA, USA). Ordinary one-way ANOVA and two-way ANOVA followed by Tukey’s test were used. *p* values below 0.05 were considered statistically significant.

### 2.9. Preparation of Washed Platelets and Immunoblotting

PRP samples were washed with ACD-Tyrode modified buffer (22.0 g/L citric acid, trisodium salt, dihydrate; 7.3 g/L citric acid, anhydrous; and 24.5 3 g/L D-(+)-Glucose, 134 mmol/L de NaCl, 2,9 mmol/L de KCl, 0,34 mmol/L de Na_2_HPO_4_, 12 mmol/L NaHCO3, 1 mmol/L de MgCl_2_ and HEPES 20 mmol/L, pH 7,4; Merck Life Science S.L.U, Madrid, Spain) and centrifugated at 1000× *g* 15 min. Prostacyclin I_2_ (PGI_2_) was added in every step to avoid platelet activation [100].

Washed platelets (~1 × 10^9^ platelets/mL) were resuspended in Tyrode modified buffer and were lysed in ice with an in-house Buffer Lysis containing protease inhibitors (Complete™ Protease Inhibitor Cocktail, Merck, Madrid, Spain). Cells transfected with the plasmids (~1 × 10^6^ cells/mL) were resuspended in PBS and lysed following the same protocol. All samples were reduced with LB-SDS solutions. Proteins were separated by 8% SDS–PAGE for 60 min and transferred to polyvinylidene fluoride membranes (Millipore, Billerica, MA, USA, Merck).

Standard Western blotting procedures were used [101]. Membranes were incubated with primary antibodies anti-P2Y12 receptor (Merck Life Science, Rabbit, #ABS1487-25UG, 1:500), anti-TBXAS1 (Cayman, Rabbit, #160715, 1:500), anti-GPVI IG5 (gently provided by Dr Elizabeth Gardiner, Melbourne, Australia Rabbit, 1:1000), anti-GPVI tail (also from Dr Elizabeth Gardiner, Rabbit 1:1000), anti-FcRI-γ (Santa Cruz Biotechnology, Dallas, TX, USA, Mouse, sc-390221, 1:1000), anti-DYKDDDK Tag (Cell Signaling Technology, Rabbit, #14793 1:1000) and anti-β actin-HRP (Merck Life Science, Rabbit cat# A3854, 1:10,000) followed by secondary horseradish peroxidase–conjugated goat anti-rabbit and anti-mouse IgG antibody (Merck Life Science, 1:10,000 and 1:5000, respectively).

Washed platelet samples were stimulated either with PBS or 3 and 10 μg/μL of CRP for 30 min at 37 °C, 500 × rpm in a shaker. Reactions were stopped, adding Buffer lysis solution following the exact procedure described for platelet samples. Membranes were incubated with primary antibodies anti-Phospho SYK (Tyr525/526) (Cell Signaling Technology, Danvers, MA, USA, rabbit, #2710, 1:1000), anti-Phospho-LAT (Tyr220) (Cell Signaling Technology, Rabbit #3584, 1:200), anti-Syk (4D10) (Santa Cruz Biotechnology, Mouse, sc-1240, 1:200), anti-LAT(B-3) (Santa Cruz Biotechnology, Mouse, sc-373706, mouse, 1:1000) followed by secondary horseradish peroxidase–conjugated goat anti-rabbit and anti-mouse IgG antibody (Merck Life Science, 1:5000). Proteins were detected by chemiluminescence (ECL prime; GE Healthcare, Madrid, Spain).

## 3. Results

### 3.1. Patient Clinical Descriptions and Molecular Diagnosis

Six female patients from four unrelated families with suspected IPFD were recruited. All of them presented normal platelet counts and mean platelet volume. Conversely, all cases had a long-standing history of moderate bleeding (ISTH –BAT scores between 4 and 8) (Table 1). None of them presented alterations in coagulation parameters or in von Willebrand factor, nor other appreciable clinical complications.

**Figure 3:**
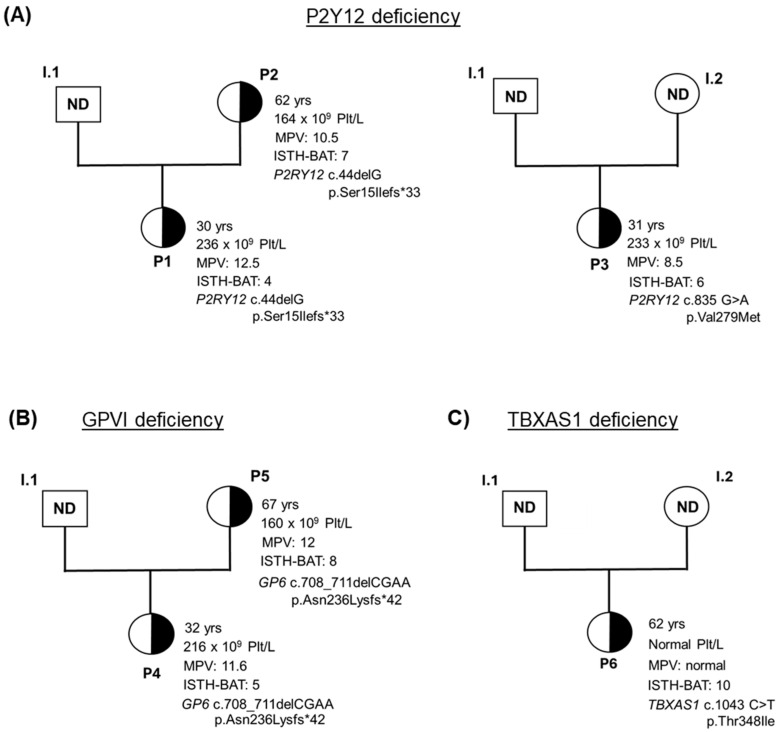
Family pedigrees from the patients involved in the study. (**A**) Pedigrees of two unrelated families with P2Y12 deficiency. (**B**) Pedigree of a family with GPVI deficiency. (**C**) Pedigree of a family with TBXAS1 deficiency. Relevant clinical information is highlighted in all probands. MPV: mean platelet volume. ND: not determined.

Patient’s DNA analysis identified different candidate heterozygous variants (Table 1). A novel nonsense variant (c.44delG [p.Ser15Ilefs*33]) was found in *P2RY12* (NM_022788.5) both in patient 1 (P1) and patient 2 (P2), located at the beginning of the protein, while patient 3 (P3) presented a novel missense variant (c.835 G>A [p.Val279Met]) located closed to a highly conserved residue involved in ADP binding (Figure 2A and Figure 3A). According to the ACMG/AMP (American College of Medical Genetics and Genomics/Association for Molecular Pathology) criteria, these variants were classified as likely pathogenic and a variant of uncertain significance (VUS), respectively (Table 1).

The novel nonsense variant c.708_711delCGAA [p.Asn236Lysfs*42], located in the stalk region of *GP6* (NM_016363.5), was identified in patient 4 and patient 5 (P4, P5) (Figure 2B and Figure 3B). This variant was classified as pathogenic, according to the ACMG/AMP classification.

Finally, patient 6 (P6) was diagnosed with a novel missense variant in *TBXAS1* (NM_001061.7) (c.1043 C>T [p.Thr348Ile]) in heterozygosis, classified as VUS. It is predicted that the variant is located in the P450 domain (Figure 2C and Figure 3C).

Our genetic studies in these patients identified no likely pathogenic or pathogenic variants in other genes that are relevant to platelet function or the *VWF* gene.

### 3.2. Platelet Phenotyping

**Figure 4:**
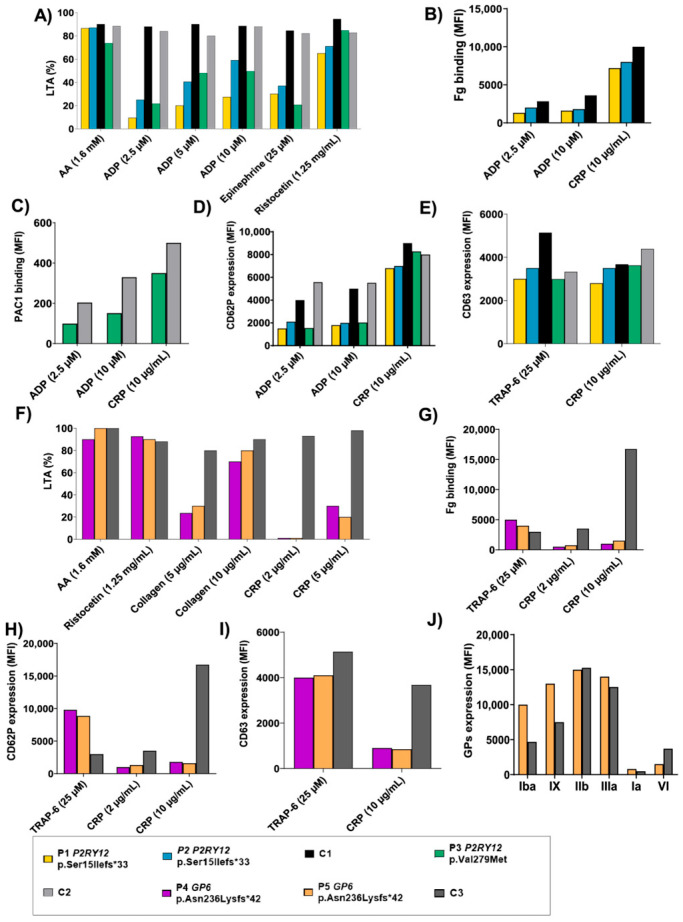
Platelet function characterization of patients with P2Y12 and GPVI deficiency. (**A**) Platelet-rich plasma aggregation profiles in response to the indicated agonists. (**B**–**E**) Flow cytometric analysis of fibrinogen and PAC1 binding, and alpha and dense granules secretion (CD62 and CD63, respectively). (**F**) Platelet-rich plasma aggregation profiles in response to the indicated agonists. % of maximal aggregation is represented. (**G**–**I**) Flow cytometric analysis of fibrinogen and PAC1 binding, and alpha and dense granules secretion (CD62 and CD63, respectively). (**J**) Flow cytometric analysis of platelet glycoproteins expression, only in the P5 patient. Unfortunately, P6, carrying the *TBXAS1* (c.1043 C>T [p.Thr348Ile]) variant, declined to be recruited for the platelet phenotype profiling.

Initial laboratory assessment of P1, P2, and P3 showed impaired platelet aggregation upon stimulation with different agonists, mainly with ADP, even at high concentrations (Figure 4A). Moreover, the three patients displayed decreased αIIbβ3 integrin activation after ADP stimulation, evaluated as fibrinogen-binding (P1, P2) or PAC1 binding (P3) (Figure 4B,C), but also reduced alpha granule secretion (Figure 4D). Dense granule expression was normal compared to the controls (Figure 4E).

On the other hand, P4 and P5 displayed a decreased platelet response to different concentrations of CRP and mild doses of collagen in platelet aggregation assays (Figure 4F). The defective response to different concentrations of CRP was also observed when evaluating fibrinogen binding, alpha granule, and dense granule secretion (Figure 4G–I).

Finally, while the expression of all major platelet glycoproteins (GPs) was normal in P1, P2, and P3, there was a decreased expression of GPVI (50%) in P5 (Figure 4J). Unfortunately, the GPs could not be evaluated in P4.

Unfortunately, P6, carrying the *TBXAS1* (c.1043 C>T [p.Thr348Ile]) variant declined to be recruited for the platelet phenotype profiling.

### 3.3. Expression of Affected Proteins

We next examined the effect of all variants in *P2RY12* and *GP6*.

**Figure 5:**
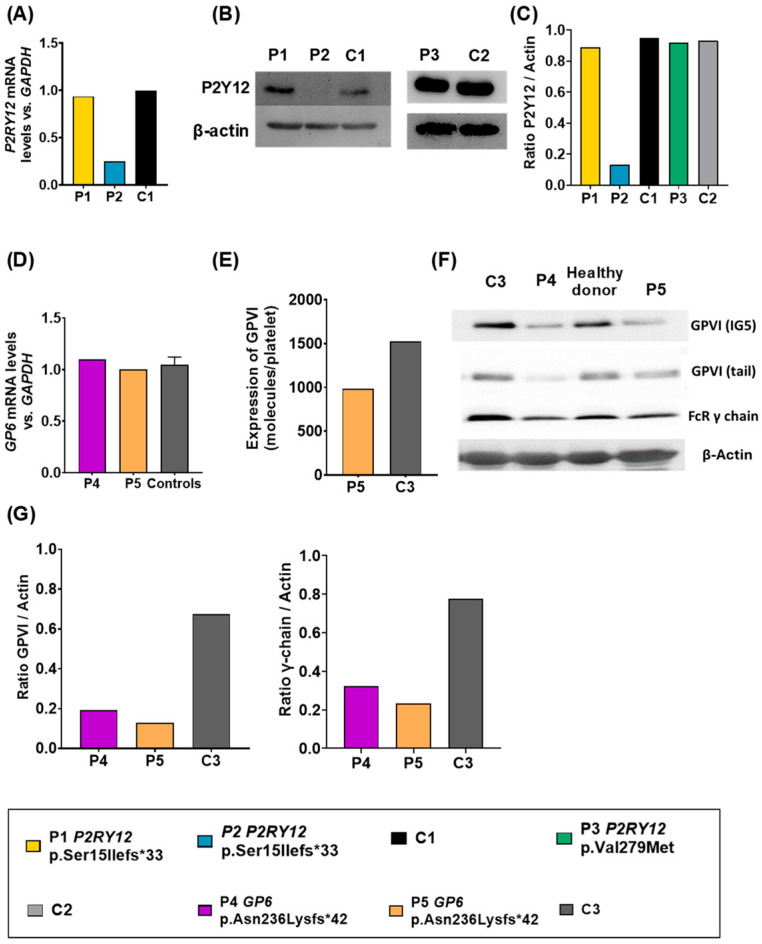
Evaluation of the effect of variants in *P2RY12* and *GP6* in patients’ platelets. (**A**) *P2RY12* mRNA expression in patient platelets relative to the GAPDH gene, used as a housekeeping gene. Unfortunately, P3 samples were not available for this assay. (**B**) Western blot determination of P2Y12 (β-actin was used as an internal control). (**C**) Densitometric analysis of protein bands of P2Y12 compared to β-Actin as a control. (**D**) *GP6* mRNA expression in patient platelets relative to the GAPDH gene, used as a housekeeping gene. (**E**) Quantitative flow cytometric analysis of GPVI expression. Unfortunately, P4 samples were not available for this assay (**F**) Western blot determination of GPVI (IG5), GPVI (tail), and FcRγ-chain (β-actin was used as an internal control). (**G**) Densitometric analysis of protein bands of GPVI (IG5) and FcRγ-chain compared to β-Actin as a control. Original Western blot images can be found in Figure S1.

P1, carrying the *P2RY12* c.44delG [p.Ser15Ilefs*33] had normal mRNA expression levels compared to the controls, whereas P2, carrier of the same variant, presented decreased levels of *P2RY12* mRNA (Figure 5A). Unfortunately, *P2RY12* mRNA levels could not be evaluated in P3.

Interestingly, immunoblotting of P2Y12 confirmed normal protein expression of the receptor in P1, but severely reduced levels in P2, while in P3, carrier of the missense variant c.835 G>A [p.Val279Met], we observed normal expression levels of the receptor (Figure 5B,C).

On the other hand, and regarding patients with GPVI defects, we observed that P4 and P5, carriers of the frameshift variant c.708_711delCGAA [p.Asn236Lysfs*42] presented normal mRNA expression levels compared to the controls (Figure 5D). However, quantitative flow cytometry assays showed decreased levels of GPVI molecules (50%) in P5 compared to the control (Figure 5E), as expected due to the creation of a premature stop codon in heterozygosis. P4 samples were not available for this test. This deleterious defect was also proven in immunoblotting assays in both patients (Figure 5F,G), since we detected a 50% reduction in levels with respect to the controls. Decreased levels of FcRγ-chain compared to the controls were also detected in P4 and P5 (Figure 5F,G). These results suggest that reduced levels of GPVI affect the expression of the γ chain.

### 3.4. Defects in P2Y12 and GPVI Signaling Pathways

**Figure 6:**
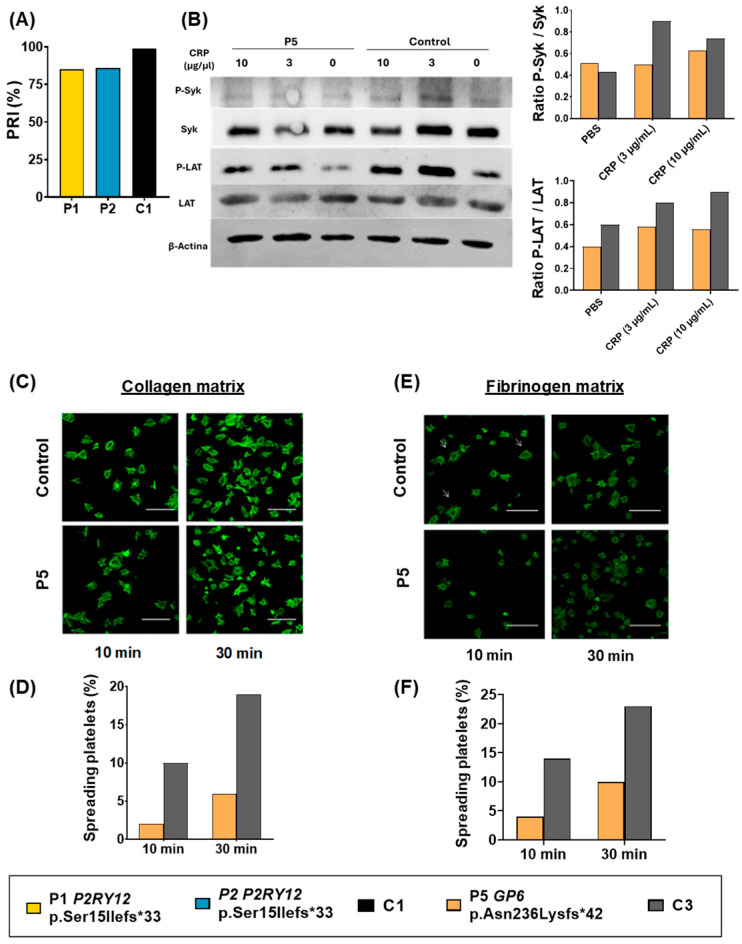
Evaluation of P2Y12 and GPVI signaling pathways in patients’ platelets. (**A**) VASP phosphorylation assessment expressed by platelet reactivity index (PRI) and determined by the VASP kit PLT VASP/P2Y12 (Biocytex, Stago, Asnières, France). (**B**) Western blot determination and densitometric analysis of P-SYK, SYK, P-LAT, and LAT expression in washed platelets stimulated with CRP 0–10 μg/μL. β-actin was used as an internal control. P4 samples were not available for this analysis. Original Western blot images can be found in Figure S1. (**C**) Immunofluorescence analysis of spreading platelets on collagen-coated coverslips incubated for 10–30 min. Platelets were labeled with fluorescein isothiocyanate-phalloidin (green). (**D**) Quatification of the spreading platelets on collagen. (**E**) Immunofluorescence analysis of spreading platelets on fibrinogen-coated coverslips incubated for 10–30 min. Platelets were labeled with fluorescein isothiocyanate-phalloidin (green). (**F**) Quantification of the spreading platelets on fibrinogen. Unfortunately, P4 samples were not available for this analysis.

Platelet VASP phosphorylation, which is a Secondary messenger in the P2Y12 pathway, was evaluated in P1 and P2 by using a PLT VASP/P2Y12 kit (Biocytex, Stago). We observed that both patients presented slightly reduced levels of platelet VASP phosphorylation in response to ADP (Figure 6A). P3 samples were not available for this test.

We also evaluated the GPVI downstream signaling cascade. Tyrosine phosphorylation of P-LAT and P-SYK was decreased in P5 compared to the control (Figure 6B). Thus, decreased expression of both GPVI and FcRγ-chain could explain platelet dysfunction in response to specific GPVI agonists.

Moreover, we also observed reduced platelet adhesion to a collagen matrix (Figure 6C,D) and fibrinogen matrix (Figure 6E,F). Further studies evaluating GPVI function as a fibrinogen and fibrin receptor will be required.

### 3.5. Disease Models in Cell Lines

We assessed the deleterious effect of all *P2RY12*, *GP6,* and *TBXAS1* variants in a cell line model. Equivalent concentrations of DYKDDDD-tagged-WT cDNA or DYKDDDK-tagged variants cDNA were transiently transfected into HEK293T cells and HEK293 COX1 (only for the *TBXAS1* variant).

**Figure 7:**
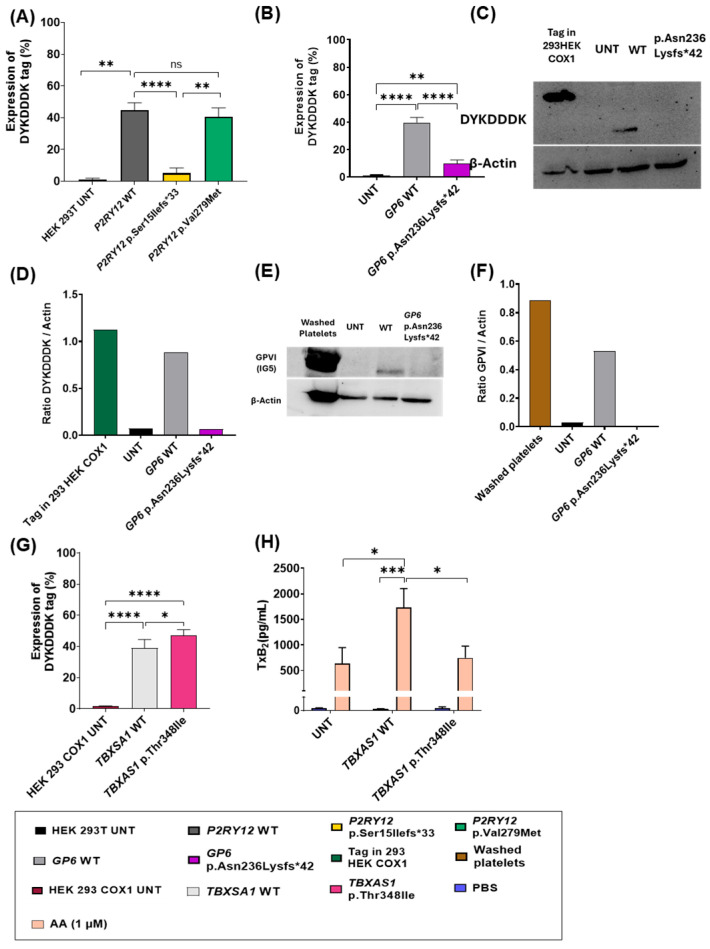
Characterization of the *P2RY12*, *GP6,* and *TBXAS1* variants in a cellular model. (**A**) Flow cytometric determination of DYKDDDK expression in HEK 293 T cells untransfected (UNT) and cells transfected with wild-type *P2RY12* and mutant p.Ser15Ilefs*33 and p.Val279Met vectors; N = 4. (**B**) Flow cytometric determination of DYKDDDK expression in HEK 293 T cells untransfected (UNT) and cells transfected with wild-type *GP6* and mutant p.Asn236Lysfs*42 vectors; N = 4. (**C**) Western blot determination of DYKDDDK expression in 293HEK COX1 cells as a control and 293 T HEK cells untransfected (UNT) and transfected with wild-type GPVI and with p.Asn236Lysfs*42 vectors. β-actin was used as an internal control. (**D**) Densitometric analysis of protein bands of DYKDDDK compared to β-Actin as a control; N = 1. (**E**) Western blot determination of GPVI expression in 293 T HEK cells untransfected (UNT) and transfected with wild-type GPVI and with p.Asn236Lysfs*42 vectors. β-actin was used as an internal control. (**F**) Densitometric analysis of protein bands of GPVI compared to β-Actin as a control. N = 1 (**G**) Flow cytometric determination of DYKDDDK expression in HEK 293 T cells untransfected (UNT) and cells transfected with wild-type *TBXAS1* and mutant p.Thr348Ile vectors;. N = 4. (**H**) TxB_2_ levels (ng/mL) in supernatants of cell stimulation reactions from the WT and mutant were measured by ELISA. N = 4. Statistical analysis one-way ANOVA was performed in flow cytometric assays. Statistical analysis two-way ANOVA followed by Tukey’s test, was performed in the ELISA assay. *: *p* ≤ 0.05, **: *p* ≤ 0.01, ***: *p* ≤ 0.001, ****: *p* ≤ 0.0001. Original Western blot images can be found in Figure S1.

Total expression of DYKDDDK-tagged-*P2RY12* p.Ser15Ilefs*33 levels were decreased compared to the wild-type protein (Figure 7A). Conversely, DYKDDDK-tagged-*P2RY12* p.Val279Met expression levels were normal, as expected considering that it is a missense variant (Figure 7A).

We also confirmed decreased levels of DYKDDDK-tagged-*GP6* p.Asn236Lysfs*42 evaluated by flow cytometry (Figure 7B). Moreover, immunoblotting revealed the absence of bands for DYKDDDK and GPVI in the transfected GPVI p.Asn236Lysfs*42 variant in HEK 293T, confirming its deleterious effect (Figure 7C–F). HEK293 cell line with a stable transfection of DYKDDK-tagged-COX1 with a molecular weight of 70kDa was used as a control (Figure 7C, first lane).

Finally, we detected normal levels of expression of DYKDDDK-tagged-TBXAS1 p.Thr348Ile by flow cytometry (Figure 7G), suggesting that the missense variant in the patient does not affect the TBXAS1 protein levels.

Next, 293 HEK COX1 cells transfected with the wild-type and *TBXAS1* p.Thr348Ile variant were stimulated with either PBS or AA (1 μM). This in-house cell line model has a stable transfection of the COX1 protein, which is required for the production of thromboxane. Total levels of TxB_2_ were decreased in TBXAS1 p.Thr348Ile transfected cells compared to the control after AA stimulation (Figure 7H). This demonstrates that the missense variant in TBXAS1 impairs TxA_2_ production in response to AA.

## 4. Discussion

IPFDs are heterogeneous in severity, mechanisms, and frequency, and present a rare prevalence [3,15]. As such, diagnosis of IPFD is challenging, as the correlation between platelet phenotype and genetic variants is often complex and not fully understood [6]. This study expands the current knowledge on P2Y12, GPVI, and TBXAS1-related disorders by reporting the effect of four novel variants in six patients from four unrelated pedigrees.

Regarding P2Y12-associated disease, we observed a somewhat different landscape in the three patients. The variant *P2RY12* p.Ser15Ilefs*33, found in P1 and P2, associates with different P2Y12 levels in both patients, since decreased mRNA and protein levels were found in P2 compared to both P1 and control. These results are surprising, as they suggest a different functional platelet phenotype in carriers of the same variant. However, this has been previously reported in other IPDs. Our group previously reported a family of six members carrying the TPM4 c.322C>T [p.Gln108*] variant, where the proband displayed lower TPM4 protein levels than the rest of her relatives [103]. All these results suggest that there are many factors, apart from the variant of study, that can modify the phenotype of patients, and it is becoming increasingly evident that monogenic diseases, such as those causing IPDs, may have a polygenic nature [104]. Of note, the *P2RY12* p.Ser15Ilefs*33 variant in the cellular model is associated with a reduction in the protein levels, as we observed in P2. These results suggest that the variant generates a premature stop codon in heterozygosity, which is associated with a 50% reduction in protein levels, but that P1 has some compensatory mechanism that makes its levels higher.

On the other hand, patient 3, who is a heterozygous carrier of the c.835 G>A [p.Val279Met] in *P2RY12*, displayed normal protein levels. This is expected considering that most of the missense variants do not affect the protein levels, but it does affect the function. Remarkably, all these results were validated in the cellular model. However, further functional studies are needed to investigate the pathogenic mechanisms of both variants.

In this paper, we have also demonstrated the deleterious effect of the variant *GP6* p.Asn236Lysfs*42 in the heterozygosity status. We observed 50% expression of GPVI protein levels, as expected for a nonsense variant. Noteworthy, we have also reported decreased levels (50%) of FcRγ-chain.

It has been previously described in the literature that the association of GPVI and FcRγ-chain in the membrane [39,105]. To date, decreased levels of FcRγ-chain have been detected only in the homozygote carriers of *GP6* p.Val238Serfs*5 variant [66]. In fact, in another study, normal levels of FcRγ-chain were reported in the patient with compound heterozygous *GP6* p.Arg58Cys and *GP6* p.Gly121Serfs*12 extracellular domains variants [67]. Noteworthy, the patient displayed a qualitative GPVI deficiency, as demonstrated by the presence of a 58kDa band corresponding to the protein. Meanwhile, our variant truncates the transmembrane region of the protein. This region is crucial for the interaction with FcRγ-chain via a salt bridge between these proteins [50]. Consequently, these results justify the decrease in FcRγ-chain since only 50% of GPVI could be detected in the membrane.

Here, we have also shown impaired phosphorylation levels of GPVI downstream signaling mediators (P-SYK and P-LAT), which suggests that the functional impairment in these patients may be caused by a defect in these signaling pathways. Of note, none of the previous studies concerning inherited GPVI disorder have performed downstream signaling analysis. Thus, there is a wide field of study in this area and disorder.

Furthermore, we have also confirmed decreased binding of spreading platelets to a fibrinogen matrix. As mentioned before, GPVI can act as a receptor of different ligands, including fibrinogen, furthering GPVI’s role in thrombus growth [47]. This ligand can interact with both monomeric and dimeric GPVI. However, dimeric clustering of GPVI increases the receptor affinity for fibrinogen [106]. Decreased expression of the receptor due to the deleterious effect of the *GP6* p.Asn236Lysfs*42 variant could lead to decreased clustering. Thus, this triggers impaired spreading of platelets to the matrix. This phenomenon was confirmed in Chilean probands who are homozygous carriers of the *GP6* p.Val238Serfs*5 variant [47].

Finally, regarding the novel *TBXAS1* p.Thr348Ile variant, we have demonstrated that the presence of this variant impairs TxA_2_ production in a cell line model. Interestingly, flow cytometry analysis of DYKDDDK expression confirmed normal levels of the mutated TBXAS1 protein. We have not been able to verify the protein levels of TBXAS1, and we cannot rule out the possibility of protein instability due to the variant, as reported in the literature for other missense variants [107].

Unfortunately, the study of this variant has been limited, as we have not been able to functionally characterize the patient carrying the variant. Thus, we could not validate the impairment of TxA_2_ production observed in a cell line model. However, this patient carrying the *TBXAS1* p.Thr348Ile variant is the first described in the literature with a history of significant bleeding and without the classic characteristics of Ghosal syndrome. These results suggest that heterozygous variants in TBXAS1 could cause a mild clinical condition, where bleeding could be present due to platelet dysfunction in the arachidonic acid and thromboxane signaling pathway, while homozygous variants cause a more severe clinical condition.

Therefore, the study of these variants expands the genetic landscape of P2Y12, GPVI, and TBXAS1 deficiencies. We demonstrate for the first time that *P2RY12* heterozygous variants associate with reduced protein levels, and they can also lead to bleeding in patients. *GP6* p.Asn236Lysfs*42 further confirms the importance of GPVI expression for the FcRγ-chain expression. And finally, *TBXAS1* variants in heterozygosis can lead to bleeding diathesis.

## 5. Conclusions

In the current study, we have described two new variants for the P2Y12 receptor, and a new variant for GPVI and TBXAS1.

We demonstrated that *P2RY12* p.Ser15Ilefs*33 and p.Val279Met variants, in heterozygosis, lead to bleeding and platelet dysfunction in response to ADP. Apart from the qualitative defect in P2Y12, we also observed that the p.Val279Met missense variant did not have an effect on the receptor expression. Interestingly, the p.Ser15Ilefs*33 variant is associated with lower protein levels, as seen in P2 and in the cell model. The mechanisms by which P1 has higher levels are unknown.

We demonstrated that p.Asn236Lysfs*42 in *GP6*, in heterozygous status, leads to platelet dysfunction in response to CRP and collagen. This variant is the first one described that truncates the transmembrane region of the glycoprotein, as well as decreases both GPVI and FcRγ-chain in heterozygosis status.

Finally, we confirmed in the cell line model that the p.Thr348Ile variant triggers decreased levels of TBX_2_ in the transfected cell line model. Our patient did not report other complications, apart from the bleeding diathesis clinical history.

## Figures and Tables

**Table 1:** Clinical characteristics of patients with suspected IPFD, recruited by GEAPC. W: woman, VUS: variant of uncertain significance.

	P1 P2Y12 Deficiency	P2 P2Y12 Deficiency	P3 P2Y12 Deficiency	P4 GPVI Deficiency	P5 GPVI Deficiency	P6 TBXAS1 Deficiency
Sex	W	W	W	W	W	W
Age (years old)	30	62	31	32	67	61
Platelet count (×10^9^/L)	236	164	233	216	160	Unknown levels, but in normal range
Mean Platelet Volume (fL)	12	12.5	8.5	11.6	12	Unknown levels, but in normal range
Bleeding score (ISTH-BAT)	4	7	6	5	8	10
Type of bleeding	Moderate bleeding diathesis	Puerperal bleeding (2 pregnancies) post-dental procedures	Moderate bleeding diathesis	Moderate bleeding diathesis	Moderate bleeding diathesis	Moderate bleeding diathesis
Variant	*P2RY12* c.44delG [p.Ser15Ilefs*33]	*P2RY12* c.44delG [p.Ser15Ilefs*33]	*P2RY12* c.835 G>A [p.Val279Met]	*GP6* c.708_711delCGAA [p.Asn236Lysfs*42]	*GP6* c.708_711delCGAA [p.Asn236Lysfs*42]	*TBXAS1* c.1043 C>T [p.Thr348Ile]
Variant status	Heterozygous	Heterozygous	Heterozygous	Heterozygous	Heterozygous	Heterozygous
Minor allele frequency (MAF) gnoMAD	Not reported	Not reported	0.00009559	0.0000962	0.0000962	0.0000159
Variant classification (ACMG)	Likely pathogenic	Likely pathogenic	VUS	Pathogenic	Pathogenic	VUS

## Data Availability

The original contributions presented in this study are included in the article/Supplementary Material. Further inquiries can be directed to the corresponding author.

## Supplementary Materials

The following supporting information can be downloaded at: https://www.mdpi.com/article/10.3390/biom15121639/s1, Figure S1: Original Western blot images. Table S1: *P2YRY12* variants in patients with inherited P2Y12 defects previously described in literature. ND: not determined; Table S2: *GP6* variants in patients with inherited GPVI defects previously described in literature. ND: not determined; Table S3: *TBXAS1* variants in patients with inherited TBXAS1 defects previously described in literature. ND: not determined.

## Abbreviations

AA	Arachidonic acid
AC	Adenylate cyclase
ACMG/AMP	American College of Medical Genetics and Genomics/Association for Molecular Pathology
ACS	Acute coronary syndrome
ADAM10	Disintegrin and metalloproteinase domain-containing protein 10
ADP	Adenosine diphosphate
ATCC	American Type Culture Collection
cAMP	Cyclic adenosine monophosphate
CLEC-2	C-type lectin domain family 2
COX1	Cicloxigenase-1
COX2	Cicloxigenase-2
cPLA2α	Cytosolic phospholipase A_2_
CRP	Collagen-related-peptide
DAG	Diacylglycerol
DIGEVAR	Discovering Genetic Variants
DMEM	Dulbecco’s modified Eagle’s medium
DNA	Deoxyribonucleic acid
cDNA	Complementary DNA
EDTA	Ethylenediaminetetraacetic acid
EL	Extracellular loop
ELISA	Enzyme-Linked Immunosorbent Assay
FBS	Fetal bovine serum
FcRγ-chain	Fc receptor-γ-chain
FcγRIIA	Low-affinity IgG receptor
Fyn	Tyrosine-protein kinase Fyn
Gαi2	Gi protein subunit α2
Giβγ	G protein subunit βγ
GHDD	Ghosal hematodiaphyseal dysplasia
GP	Glycoprotein
GPCR	G-protein-coupled receptor
GPIa	Integrin α2
GPIbα	Glycoprotein Ib (GPIb), also known as CD42b
GPIIb	Integrin αIIb, also known as CD41
GPIIIa	Integrin β, also known as CD61
GPIX	Glycoprotein IX, also known CD49b
GPVI	Platelet glycoprotein VI
*GP6*	Platelet glycoprotein VI gene
HGVS	Human Genome Variation Society
HTS	High-throughput sequencing
IL	Intracellular loop
IPDs	Inherited Platelet Disorders
IPFD	Inherited Platelet Function Disorders
ISTH-BAT	International Society on Thrombosis and Haemostasis Bleeding Assessment Tool
IT	Inherited thrombocytopenias
ITAM	Immunoreceptor tyrosine-based activation motifs
KO	Knock-out
LAT	Linker for activation of T-cells family member 1 protein
LB-SDS	Loading buffer and
LCR	Leukocyte receptor cluster
LTA	Light transmission aggregometry
Lyn	Tyrosine-protein kinase Lyn
MAF	Minor allele frequency
MFI	Median Fluorescence Intensity
MPV	Mean platelet volume
MUT	Mutant
NSAIDs	Nonsteroidal anti-inflammatory drugs
*P2RY12*	Purinergic Receptor P2Y12 gene
P2Y12	Purinergic Receptor P2Y12 protein
PBS	Phosphate-Buffered Saline
PDZ	Postsynaptic density 95/disk large/zonula occludens-1
PFA	Paraformaldehyde
PFA-100	Platelet Function Analizer-100 assay
PGE2	Prostaglandin E2
PGH2	prostaglandin H2
PGI2	Prostacyclin I2
PI3K	Phosphoinositide 3-kinase
PKA	Protein kinase A
PKC	Protein kinase C
PLCγ2	Phospholipase C
PRD	Proline-rich domain
PRI	Platelet Reactivity Index
PRP	Platelet rich plasma
PPP	Platelet poor plasma
SDS-PAGE	Sodium dodecyl sulfate-polyacrylamide gel electrophoresis
SLP-76	Lymphocyte cytosolic protein 2
SNP	Single nucleotide polymorphism
Syk	Tyrosine-protein kinase Syk
SVs	Structural variants
*TBXAS1*	Thromboxane synthase gene
TBXAS1	Thromboxane synthase protein
TRAP	Thrombin receptor-activating peptide
TxA2	Thromboxane A2
TxB2	Thromboxane B2
VASP	Vasodilator-stimulated phophoprotein
VCF	Variant calling files
VSMC	Vascular smooth muscle cells
VUS	Variant of uncertain significance
WT	Wild-type
