# Supplementary material for: Characterization of Novel Variants in P2YRY12, GP6 and TBXAS1 in Patients with Lifelong History of Bleeding
Source: Biomolecules. 2025 Nov 21;15(12):1639. doi: 10.3390/biom15121639 (PMC12730773; doi:10.3390/biom15121639)
Supplement: Supplementary file 1 [file biomolecules-15-01639-s001.zip › biomolecules-3972089-supplementary.pdf]

Figure S1: Original Western blot images

WB shown in Figure 5B

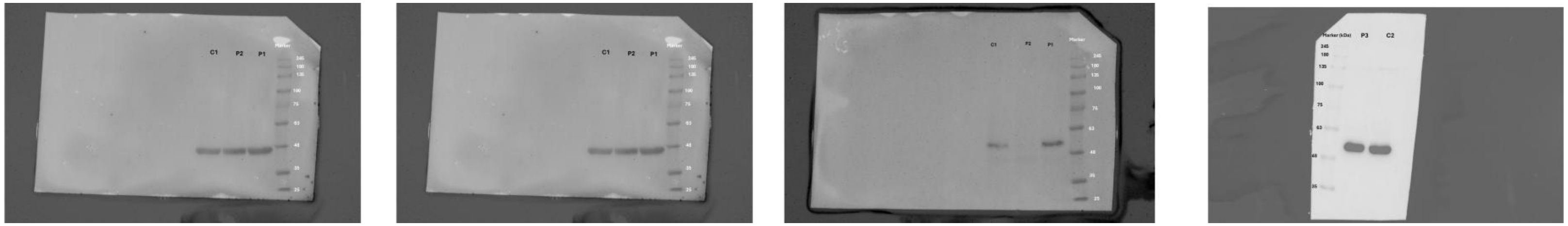

WB shown in Figure 5F

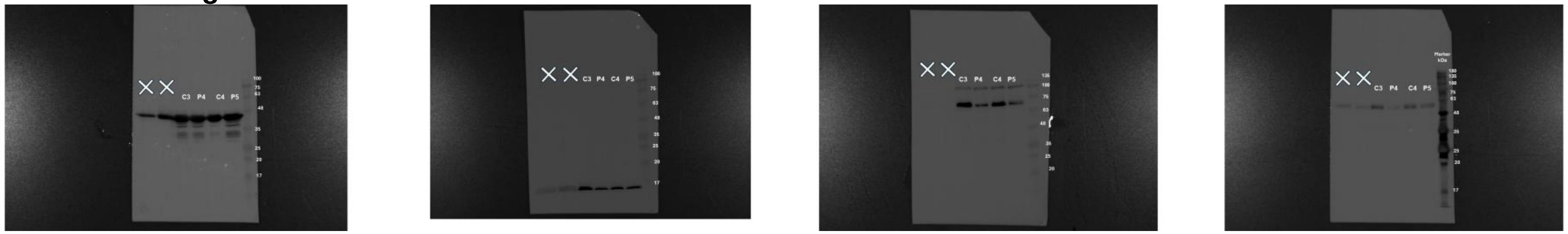

WB shown in Figure 6B

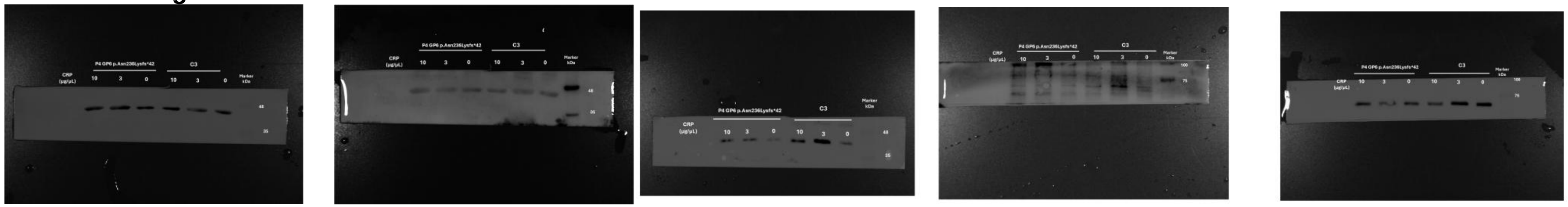

WB shown in Figure 7C

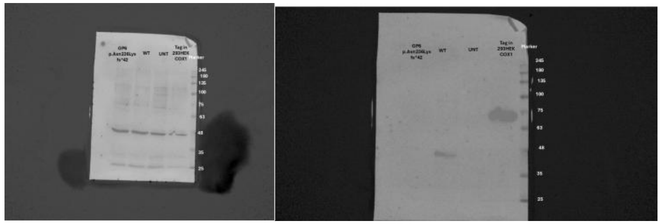

WB shown in Figure 7E

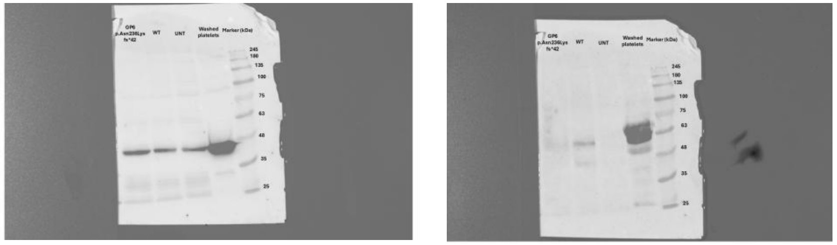

Supplemental Table S1. ***P2YRY12*** **variants in patients with inherited P2Y12 defects previously described in literature.** ND: not determined

Variant	Status	ADP Platelet response	P2Y12 receptor expression	Bleeding	Reference
**p.Met1Arg**	Homozygous	Impaired	Decreased (cell line model only)	Mild bleeding	[1]
**p.Gly12Valfs*36**	Homozygous	Impaired	Decreased	Mild bleeding	[2]
**p.Gln98Lysfs*20**	Homozygous	Impaired	Decreased	Mild bleeding	[3]
**p.Arg122Cys**	Heterozygous	Impaired	Normal	Mild bleeding	[4]
**p.Arg122His**	Heterozygous	Impaired	Normal	Mild bleeding	[5]
**p.Thr126Aspfs*34**	Hemizygous	Impaired	Decreased	Mild bleeding	[6,7]
	Homozygous/	Impaired	ND	Mild bleeding	[8]
	Compound heterozygous#	Impaired	ND	Mild bleeding	
**p.Lys174Glu**	Heterozygous	Impaired	Normal	ND	[9]
**p.His187Gln**	Homozygous	Impaired	Normal	Mild bleeding	[4]
**p.Val207Ala**	Heterozygous	Impaired	Normal (cell line model only)	ND	[10]
**p.Ile240Tyrfs*29**	Heterozygous	Impaired	Decreased	Mild bleeding	[11]
**p.Arg256Gln + Arg265Trp**	Compound heterozygous	Impaired	Normal	Mild bleeding	[12]
**p.Pro258Thr**	Heterozygous	Impaired	Normal	Mild bleeding	[5,13]
**p.Arg265Pro**	Heterozygous	Impaired	Normal	Lifelong bleeding history	[14,15]
**p.Pro341Ala**	Heterozygous	Impaired	Normal	Mild bleeding	[15]

Supplemental Table S2. *GP6* variants in patients with inherited GPVI defects previously described in literature. ND: not determined

Variant	Status	Collagen platelet response	GPVI receptor expression	Bleeding	Reference
**ND**	ND	Impaired	Absent	Mild beeding (epistaxis, menorrhagia).<br>Family history of bleeding	[16]
		Impaired	10%	Lifelong mild bleeding.<br>Required transfusion after surgery.<br>No familial history	[17]
**p.Gln49_Gly53del**	Heterozygous	ND	ND	ND	[18]
**p.Gln49_Gly53del + p.Ser175Asn**	Compound heterozygous	Impaired	Decreased	Ecchymoses, epistaxis, menorrhagia	[18]
**p.Arg58Cys**	Heterozygous	Normal	50% decreased	No	[19]
**p.Arg58Cys + p.Gly121Serfs*12**	Compound heterozygous	Impaired	Absent	Ecchymoses during childhood	[19]
**p.Gly121Serfs*12**	Heterozygous	Normal	50% decreased	No	[19]
**p.Ser175Asn**	Heterozygous	Slightly decreased	Normal	No	[18]
**p.Val238Serfs*5**	Heterozygous	Normal	Normal	No	[20]
	Homozygous	Impaired	Decreased	Mild bleeding in all except one	

Supplemental Table S3. *TBXAS1* variants in patients with inherited TBXAS1 defects previously described in literature. ND: not determined

Variant	Status	Platelet function	Ghosal dysplasia	Bleeding	Reference
**ND**	ND	Impaired in response to arachidonic acid, ADP and collagen.<br>TBX2 production impaired	ND	Yes (familial bleeding history)	[21]
		ND	Yes (thrombocytopenia)	No	[22]
		ND	Yes (thrombocytopenia)	No	[23]
		ND	Yes (thrombocytopenia)	No	[24]
		ND	Yes	No	[25]
		ND	Yes	No	[26]
		ND	Yes (thrombocytopenia)	No	[27]
		ND	Yes	No	[28]
		ND	Yes	No	[29]
**p.Leu83Pro**	Homozygous	Impaired response to arachidonic acid	Yes	No	[30]
**p.Ile89Thr + p.Leu330Pro**	Compound heterozygous	Impaired in response to arachidonic acid	Yes	No	[31]
**p.Ala195Leufs*12 + p.Gly474Trp**	Compound heterozygous	Impaired response to arachidonic acid	Yes	ND	[32]
**p.Glu250***	Homozygous	ND	Yes (thromboctypenia)	No	[33]
**p.Arg287***	Homozygous	ND	Yes (thrombocytopenia)	No	[34]
**p.Arg412Gln**	Homozygous	ND	Yes	No	[33,35]
**p.Arg413Glu**	Homozygous	Impaired response to	Yes	No	[30,36]

		arachidonic acid			
**p.Arg413Gln**	Homozygous	Impaired platelet aggregation response to arachidonic acid. Decreased levels of TXA2 production	Yes	No	[30,34,35,37]
**p.Arg459Gln**	Homozygous	ND	Yes	No	[38,39]
**p.Arg459Gln + p.Lys42Thrfs*47**	Compound heterozygous	ND	Yes	No	[40]
**p.Gly482Trp**	Homozygous	Impaired in response to arachidonic acid	Yes	No	[30]
**p.Leu488Pro**	Homozygous	Impaired in response to arachidonic acid	Yes	No	[30]

References

1. Shiraga, M.; Miyata, S.; Kato, H.; Kashiwagi, H.; Honda, S.; Kurata, Y.; Tomiyama, Y.; Kanakura, Y. Impaired Platelet Function in a Patient with P2Y12 Deficiency Caused by a Mutation in the Translation Initiation Codon. *Journal of Thrombosis and Haemostasis* **2005**, *3*, 2315–2323, doi:10.1111/j.1538-7836.2005.01554.x.
2. Cattaneo, M. Molecular Defects of the Platelet P2 Receptors. *Purinergic Signalling* **2011**, *7*, 333–339, doi:10.1007/S11302-011-9217-Z,.
3. Cattaneo, M.; Lecchi, A.; Randi, A.M.; McGregor, J.L.; Mannucci, P.M. Identification of a New Congenital Defect of Platelet Function Characterized by Severe Impairment of Platelet Responses to Adenosine Diphosphate. *Blood* **1992**, *80*, 2787–2796, doi:10.1182/blood.v80.11.2787.bloodjournal80112787.
4. Patel, Y.M.; Lordkipanidzé, M.; Lowe, G.C.; Nisar, S.P.; Garner, K.; Stockley, J.; Daly, M.E.; Mitchell, M.; Watson, S.P.; Austin, S.K.; et al. A Novel Mutation in the P2Y12 Receptor and a Function-Reducing Polymorphism in Protease-Activated Receptor 1 in a Patient with Chronic Bleeding. *Journal of Thrombosis and Haemostasis* **2014**, *12*, 716–725, doi:10.1111/jth.12539.
5. Jones, M.L.; Norman, J.E.; Morgan, N.V.; Mundell, S.J.; Lordkipanidzé, M.; Lowe, G.C.; Daly, M.E.; Simpson, M.A.; Drake, S.; Watson, S.P.; et al. Diversity and Impact of Rare Variants in Genes Encoding the Platelet G Protein-Coupled Receptors. *Thrombosis and Haemostasis* **2015**, *113*, 826–837, doi:10.1160/TH14-08-0679,.
6. Cattaneo, M.; Lecchi, A.; Lombardi, R.; Gachet, C.; Zighetti, M.L. Platelets from a Patient Heterozygous for the Defect of P2CYC Receptors for ADP Have a Secretion Defect despite Normal Thromboxane A2 Production and Normal Granule Stores: Further Evidence That Some Cases of Platelet "primary Secretion Defect" Are Heterozygous for a Defect of P2CYC Receptors. *Arteriosclerosis, thrombosis, and vascular biology* **2000**, *20*, doi:10.1161/01.ATV.20.11.E101.
7. Fontana, G.; Ware, J.; Cattaneo, M. Haploinsufficiency of the Platelet P2Y12 Gene in a Family with Congenital Bleeding Diathesis. *Haematologica* **2009**, *94*, 581–584, doi:10.3324/HAEMATOL.13611,.
8. Lunghi, B.; Lecchi, A.; Santacroce, R.; Scavone, M.; Paniccia, R.; Artoni, A.; Gachet, C.; Castaman, G.; Margaglione, M.; Bernardi, F.; et al. Severe Bleeding and Absent ADP-Induced Platelet Aggregation Associated with Inherited Combined CalDAG-GEFI and P2Y12 Deficiencies. *Haematologica* **2020**, *105*, E361–E364, doi:10.3324/HAEMATOL.2019.232850,.
9. Daly, M.E.; Dawood, B.B.; Lester, W.A.; Peake, I.R.; Rodeghiero, F.; Goodeve, A.C.; Makris, M.; Wilde, J.T.; Mumford, A.D.; Watson, S.P.; et al. Identification and Characterization of a Novel P2Y12 Variant in a Patient Diagnosed with Type 1 von Willebrand Disease in the European MCMDM-1VWD Study. *Blood* **2009**, *113*, 4110–4113, doi:10.1182/BLOOD-2008-11-190850,.
10. Jones, M.L.; Norman, J.E.; Morgan, N.V.; Mundell, S.J.; Lordkipanidzé, M.; Lowe, G.C.; Daly, M.E.; Simpson, M.A.; Drake, S.; Watson, S.P.; et al. Diversity and Impact of Rare Variants in Genes Encoding the Platelet G Protein-Coupled Receptors. *Thromb Haemost* **2015**, *113*, 826–837, doi:10.1160/TH14-08-0679.
11. Hollopeter, G.; Jantzen, H.M.; Vincent, D.; Li, G.; England, L.; Ramakrishnan, V.; Yang, R.B.; Nurden, P.; Nurden, A.; Julius, D.; et al. Identification of the Platelet ADP Receptor Targeted by Antithrombotic Drugs. *Nature* **2001**, *409*, 202–207, doi:10.1038/35051599,.
12. Cattaneo, M.; Zighetti, M.L.; Lombardi, R.; Martinez, C.; Lecchi, A.; Conley, P.B.; Ware, J.; Ruggeri, Z.M. Molecular Bases of Defective Signal Transduction in the Platelet P2Y12 Receptor of a Patient with Congenital Bleeding. *Proceedings of the National Academy of Sciences of the United States of America* **2003**, *100*, 1978–1983, doi:10.1073/PNAS.0437879100,.

13. Remijn, J.A.; Ijsseldijk, M.J.W.; Strunk, A.L.M.; Abbes, A.P.; Engel, H.; Dikkeschei, B.; Dompeling, E.C.; Groot, P.G.D.; Slingerland, R.J. Novel Molecular Defect in the Platelet ADP Receptor P2Y12 of a Patient with Haemorrhagic Diathesis. *Clinical Chemistry and Laboratory Medicine* **2007**, *45*, 187–189, doi:10.1515/CCLM.2007.036,.
14. Mundell, S.J.; Rabbolini, D.; Gabrielli, S.; Chen, Q.; Aungraheeta, R.; Hutchinson, J.L.; Kilo, T.; Mackay, J.; Ward, C.M.; Stevenson, W.; et al. Receptor Homodimerization Plays a Critical Role in a Novel Dominant Negative P2RY12 Variant Identified in a Family with Severe Bleeding. *Journal of Thrombosis and Haemostasis* **2018**, *16*, 44–53, doi:10.1111/jth.13900.
15. Nisar, S.; Daly, M.E.; Federici, A.B.; Artoni, A.; Mumford, A.D.; Watson, S.P.; Mundell, S.J. An Intact PDZ Motif Is Essential for Correct P2Y12 Purinoceptor Traffic in Human Platelets. *Blood* **2011**, *118*, 5641–5651, doi:10.1182/BLOOD-2011-02-336826,.
16. Moroi, M.; Jung, S.M. Platelet Glycoprotein VI: Its Structure and Function. *Thrombosis Research* **2004**, *114*, 221–233, doi:10.1016/j.thromres.2004.06.046.
17. Borst, O.; Gawaz, M. Glycoprotein VI - Novel Target in Antiplatelet Medication. *Pharmacology and Therapeutics* **2021**, *217*, doi:10.1016/j.pharmthera.2020.107630.
18. Hermans, C.; Wittevrongel, C.; Thys, C.; Smethurst, P.A.; Van Geet, C.; Freson, K. A Compound Heterozygous Mutation in Glycoprotein VI in a Patient with a Bleeding Disorder. *Journal of Thrombosis and Haemostasis* **2009**, *7*, 1356–1363, doi:10.1111/j.1538-7836.2009.03520.x.
19. Dumont, B.; Lasne, D.; Rothschild, C.; Bouabdelli, M.; Ollivier, V.; Oudin, C.; Ajzenberg, N.; Grandchamp, B.; Jandrot-Perrus, M. Absence of Collagen-Induced Platelet Activation Caused by Compound Heterozygous GPVI Mutations. *Blood* **2009**, *114*, 1900–1903, doi:10.1182/BLOOD-2009-03-213504,.
20. Matus, V.; Valenzuela, G.; Sáez, C.G.; Hidalgo, P.; Lagos, M.; Aranda, E.; Panes, O.; Pereira, J.; Pillois, X.; Nurden, A.T.; et al. An Adenine Insertion in Exon 6 of Human GP6 Generates a Truncated Protein Associated with a Bleeding Disorder in Four Chilean Families. *Journal of Thrombosis and Haemostasis* **2013**, *11*, 1751–1759, doi:10.1111/jth.12334.
21. Mestel, F.; Oetliker, O.; Beck, E.; Felix, R.; Imbach, P.; Wagner, H.P. SEVERE BLEEDING ASSOCIATED WITH DEFECTIVE THROMBOXANE SYNTHETASE. *The Lancet* **1980**, *315*, 157, doi:10.1016/S0140-6736(80)90642-X.
22. Mondal, R.; Sil, A.; Nag, S.S.; Sabui, T. Ghosal Syndrome – Ten Years Follow-Up. *Indian Journal of Pediatrics* **2015**, *82*, 568–569, doi:10.1007/S12098-014-1654-6,.
23. Mondal, R.K.; Karmakar, B.; Chandra, P.K.; Mukherjee, K. Ghosal Type Hemato-Diaphyseal Dysplasia: A Rare Variety of Engelmann's Disease. *Indian Journal of Pediatrics* **2007**, *74*, 291–293, doi:10.1007/S12098-007-0047-5,.
24. Ciftciler, R.; Buyukasık, Y.; Saglam, E.A.; Haznedaroglu, I.C. Ghosal Hematodiaphyseal Dysplasia with Autoimmune Anemia in Two Adult Siblings. *Transfusion and Apheresis Science* **2019**, *58*, 449–452, doi:10.1016/j.transci.2019.04.027.
25. John, R.R.; Boddu, D.; Chaudhary, N.; Yadav, V.K.; Mathew, L.G. Steroid-Responsive Anemia in Patients of Ghosal Hematodiaphyseal Dysplasia: Simple to Diagnose and Easy to Treat. *Journal of Pediatric Hematology/Oncology* **2015**, *37*, 285–289, doi:10.1097/MPH.0000000000000279,.
26. Arora, R.; Aggarwal, S.; Deme, S. Ghosal Hematodiaphyseal Dysplasia—a Concise Review Including an Illustrative Patient. *Skeletal Radiology* **2015**, *44*, 447–450, doi:10.1007/S00256-014-1989-0,.
27. Kini, P.G.; Kumar, S.; Moideen, A.; Narain, A.T. Ghosal Hemato-Diaphyseal Dysplasia: A Rare Variety of Hypoplastic Anemia with Good Response to Steroid Therapy. *Indian Journal of Hematology and Blood Transfusion* **2018**, *34*, 181–182, doi:10.1007/S12288-017-0818-8,.
28. Datta, K.; Karmakar, M.; Hira, M.; Halder, S.; Pramanik, K.; Banerjee, G. Ghosal Hematodiaphyseal Dysplasia with Myelofibrosis. *Indian Journal of Pediatrics* **2013**, *80*, 1050–1052, doi:10.1007/S12098-012-0872-Z,.

29. Shakiba, M.; Shamsian, S.; Malekzadeh, H.; Yasaei, M. Ghosal Hematodiaphyseal Dysplasia: A Case Report. *Int J Hematol Oncol Stem Cell Res* **2020**, *14*, 127–129.
30. Geneviève, D.; Proulle, V.; Isidor, B.; Bellais, S.; Serre, V.; Djouadi, F.; Picard, C.; Vignon-Savoye, C.; Bader-Meunier, B.; Blanche, S.; et al. Thromboxane Synthase Mutations in an Increased Bone Density Disorder (Ghosal Syndrome). *Nature Genetics* **2008**, *40*, 284–286, doi:10.1038/NG.2007.66,.
31. Sharma, R.; Sierra Potchanant, E.; Schwartz, J.E.; Nalepa, G. Chronic Steroid-Response Pancytopenia and Increased Bone Density Due to Thromboxane Synthase Deficiency. *Pediatric Blood and Cancer* **2018**, *65*, doi:10.1002/PBC.26777,.
32. Kim, S.Y.; Ing, A.; Gong, S.; Yap, K.L.; Bhat, R. Novel Compound Heterozygous Variants of TBXAS1 Presenting with Ghosal Hematodiaphyseal Dysplasia Treated with Steroids. *Molecular Genetics and Genomic Medicine* **2021**, *9*, doi:10.1002/MGG3.1494,.
33. Uludağ Alkaya, D.; Usluer, E.; Alp Ünkar, Z.; Şeker, A.; Adaletli, İ.; Güneş, N.; Madazlı, R.; Kadıoğlu, P.; Derbent, M.; Tüysüz, B. Insights into Natural History, Phenotypic, and Molecular Spectrum in a Large Cohort of Osteosclerotic Disorders. *Calcified Tissue International* **2025**, *116*, doi:10.1007/S00223-025-01366-W,.
34. Brown, T.J.; Barrett, N.; Meng, H.; Ricciotti, E.; McDonnell, C.; Dancis, A.; Qualtieri, J.; FitzGerald, G.A.; Cotter, M.; Babushok, D.V. Nonsteroidal Anti-Inflammatory Drugs as a Targeted Therapy for Bone Marrow Failure in Ghosal Hematodiaphyseal Dysplasia. *Blood* **2023**, *141*, 1553–1559, doi:10.1182/BLOOD.2022018667,.
35. Ravikumar, D.B.; Sivasubramanian, B.P.; Thungala, S.; Srinivasan, G.; Khader, A.H.S.A.; Qadeer, H.; Panchal, V.; Venkata, V.S. Middle-Aged Women with Hematodiaphyseal Dysplasia: Ghosal Syndrome: Case Report. *Radiology Case Reports* **2024**, *19*, 4578–4582, doi:10.1016/j.radcr.2024.07.028.
36. Jeevan, A.; Doyard, M.; Kabra, M.; Daire, V.C.; Gupta, N. Ghosal Type Hematodiaphyseal Dysplasia. *Indian Pediatrics* **2016**, *53*, 347–348, doi:10.1007/S13312-016-0851-Y,.
37. Joy, P.; Yoganathan, S.; Korula, S.; Abraham, S.S.C.; Barney, A.M.; Walter, V.M.; Gibikote, S.; Danda, S. Ghosal Hematodiaphyseal Dysplasia and Response to Corticosteroid Therapy. *American Journal of Medical Genetics, Part A* **2021**, *185*, 596–599, doi:10.1002/AJMG.A.61961,.
38. Selina, A.; Kandagaddala, M.; Madhuri, V. A Recurrent Biallelic Pathogenic Variant in TBXAS1 Gene Causing Ghosal Hematodiaphyseal Dysplasia. *Indian Journal of Pediatrics* **2021**, *88*, 381–382, doi:10.1007/S12098-020-03581-9,.
39. Ong, S.G.; Ding, H.J.; Chan, M.Y.; Loh, W.K.; Mahmood, M.J. Case Report: Ghosal Hematodiaphyseal Dysplasia—A Rare Cause of Skeletal Dysplasia and Cytopenia. *International Journal of Rheumatic Diseases* **2024**, *27*, doi:10.1111/1756-185X.15220,.
40. Sudhakar, M.; Sharma, M.; Kandasamy, S.; Gummadi, A.; Rawat, A.; Vignesh, P. Novel TBXAS1 Variants in Two Indian Children with Ghosal Hematodiaphyseal Dysplasia: A Concise Report. *European Journal of Medical Genetics* **2022**, *65*, doi:10.1016/j.ejmg.2022.104498.
